# Responses in thermal tolerance and daily activity rhythm to urban stress in *Drosophila suzukii*

**DOI:** 10.1002/ece3.9616

**Published:** 2022-12-12

**Authors:** Ayame Sato, Yuma Takahashi

**Affiliations:** ^1^ Graduate School of Science and Engineering Chiba University Chiba Japan; ^2^ Graduate School of Science Chiba University Chiba Japan

**Keywords:** adaptation, Anthropocene, artificial light at night, insect, locomotor activity, spotted wing drosophila, thermal tolerance

## Abstract

Cities experience changes in abiotic factors, such as warming, increases in noise and light. These changes can lead to phenotypic changes. Several studies have revealed that altered environments change phenotypes in plants and animals in cities. However, limited studies have isolated evolutionary from nongenetic changes. Here, we analyzed the evolution of thermal tolerance and diurnal activity patterns in the urban population of the fruit pest, *Drosophila suzukii*. Urban and rural isofemale lines were reared under constant conditions. We compared the lower and upper thermal limits (CT_min_ and CT_max_, respectively), and effects of temperature exposure on the thermal limits of urban and rural populations. Common garden experiments showed that urban populations exhibit a lower CT_min_ than rural populations, suggesting genetic difference in CT_min_ among populations. On the other hand, the difference in CT_max_ between urban and rural populations was not significant. Exposure to cold temperature did not affect CT_min_ in both urban and rural populations. In contrast, exposure to hot temperature increased CT_max_ especially in urban population, suggesting that urban populations evolved in response to urban heat. We also investigated the daily activity patterns of urban and rural populations and the effect of lifelong artificial light at night on daily activity. We found that night‐time light (dim light) reduced the total amount of activity compared to dark night condition. In addition, dim light at night altered the daily rhythm of activity and increased the activity rate at night. The effect of night light on total activity was less in urban than that in rural populations, suggesting that populations in cities evolved to mitigate decreased activity under night light. Our results showed that environmental temperature and artificial light at night evolutionarily and plastically influence ecologically important traits, such as temperature tolerance and diurnal activity.

## Graphical abstract

Our study examined the evolutionary and plastic changes in thermal tolerances and diurnal activity pattern in urban populations of *Drosophila suzukii*. Results revealed that urban populations get evolved in cold tolerance and ability to improve heat tolerance and night‐time light changes diurnal activity patterns. The evolutionary responses in urban populations could contribute to the survival in the urban environments.

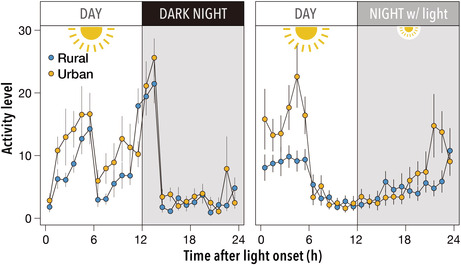

## 1 INTRODUCTION

Our planet has been dramatically urbanized. Fifty‐five percent of the world's population live in cities (United Nations, 2019), which are increasingly expanding. Urban environments are typical hotspots for human activity, leading to the alteration of several habitats. Cities experience changes in abiotic factors, such as warming (Mentaschi et al., 2022; Simwanda et al., 2019; Tran et al., 2017), increased noise (Swaddle et al., 2015), light (Duque et al., 2019; Gaston et al., 2013), and chemical pollution (Castells‐Quintana et al., 2021; Han et al., 2014), as well and biotic factors, such as habitat fragmentation and shrinking, due to the development of artificial structures (Liu et al., 2016). These changes affect the ecological dynamics of the regional populations and communities (Achury et al., 2021; Hamer et al., 2021; Hung et al., 2019). For instance, urbanization has possibly led to a recent decrease in the number of species in birds and invertebrates in these environments (Sol et al., 2017). Rapid environmental changes in urban areas have also been suggested to induce phenotypic changes in urban animals and plants (Beasley et al., 2018; Grunst et al., 2020; Su et al., 2021). Phenotypic changes can affect the survival and reproduction of organisms inhabiting urban environments, revealing that these phenotypic changes are critical for understanding population declines in urban environments and assessing the impact of urbanization on organisms at regional and global scales.

Since phenotypic changes include both genetic and plastic (i.e., nongenetic) changes, we should distinguish these two processes to disentangle the mechanisms underlying phenotypic changes occurring with urbanization. Here, genetic differences between urban and rural populations are evidence for contemporary evolution as a response against urbanization (Cheptou et al., 2008; Mueller et al., 2013; Reid et al., 2016). The observed contemporary evolution could be a result of adaptive evolution against drastic environmental changes in cities (Harpak et al., 2021) and/or stochastic drift due to habitat fragmentation in urban landscapes (Byrne & Nichols, 1999; Gortat et al., 2015; Miles et al., 2019; Mueller et al., 2018). However, plastic shift can appear as flexibility, that is, reversible and ad hoc changes in response to temporal changes in the environment (Starck & Beese, 2001), and phenotypic plasticity, that is, irreversible developmental changes depending on the environment to which the individual is exposed during ontogeny (Pfab et al., 2016; Sergio et al., 2021; Swaddle et al., 2015). In addition, phenotypes can be under a transgenerational epigenetic effect, which is governed by environments experienced by the parent or grandparent (Heath et al., 1999; Mousseau, 1998). Since a phenotype observed in the field is the sum of genetic and non‐genetic changes, common garden experiments are needed to isolate evolutionary changes in phenotypes, to quantify the relative contribution of evolutionary change and nongenetic changes to the total observed differences in phenotypes between rural and urban populations. Furthermore, to control the transgenerational epigenetic effect, it is also important to rear multiple generations in common garden environments before the experiments. In addition, non‐genetic changes can be detected by observing the phenotypes of genetically identical individuals exposed to different environments. However, in practice, it is difficult to achieve such an ideal experimental procedure in many wildlife species because of difficulties in rearing under laboratory conditions and/or their long generation time (Dahirel et al., 2019; Hall & Warner, 2018; Merckx et al., 2018; Pagliaro & Knouft, 2020; Theodorou et al., 2021).

Common environmental changes in cities include increased temperature (Li et al., 2018; Liu et al., 2015; Wang et al., 2017) and artificial light at night (ALAN) (Cinzano et al., 2001; Kyba et al., 2015). Heat in cities can differentially affect species and result in rapid thermal adaptation (Johnson & Stahlschmidt, 2020; Pagliaro & Knouft, 2020). This is particularly important for ectotherms because their development, growth, and reproduction are dependent on environmental temperatures (Atkinson, 1995; Chamaillé‐Jammes et al., 2006; Gillooly et al., 2001; Paaijmans et al., 2013). Phenotypic shifts in thermal tolerance across urbanization temperature clines have been observed (Angilletta Jr et al., 2007; Sánchez‐Echeverría et al., 2019). The shift in thermal tolerance is determined both non‐genetically and genetically. Therefore, both can contribute to thermal adaptation, together with urbanization. In contrast, ALAN can disrupt behavioral and physiological processes (Aulsebrook et al., 2020; Durrant et al., 2018; Miner et al., 2021). ALAN has been suggested to affect reproduction and phenology (Dominoni et al., 2013; McLay et al., 2017). Although light is a critical factor determining circadian rhythm, little is known about the effects of ALAN on the diurnal activity of individuals (cf. Duarte et al., 2019). Because these effects of ALAN lead to a decline in reproductive success (McLay et al., 2018; Thompson et al., 2019), urban populations can achieve adaptive evolution against ALAN. Although some studies have shown adaptive shifts in behavior linked to light (Altermatt & Ebert, 2016), it is unclear whether the shift represents adaptive evolution.

Here, we tested the evolution of thermal tolerance and diurnal activity rhythm in an urban population in Japan, as well as the effect of temperature acclimation on thermal tolerance and the effect of ALAN on diurnal activity patterns in the spotted wing fruit fly, *Drosophila suzukii*. This species is native to Japan and widely inhabits rural and urban environments. Similarly to other ectothermic species, *D. suzukii* is expected to be sensitive to environmental temperatures. In addition, *Drosophila* flies are suitable for detecting both genetic and plastic phenotypic shifts using common garden experiments because of their ease of rearing during multiple generations under laboratory conditions.

## 2 MATERIALS AND METHODS

### 2.1 Study species

We used *D. suzukii*, an invasive polyphagous fruit pest that damages berries and small stone fruits by ovipositing them. It has rapidly spread worldwide in recent years (Adrion et al., 2014; Rota‐Stabelli et al., 2013). It commonly inhabits both urban and rural habitats and occurs mainly from May to August. *D. suzukii* is a close relative of *D. melanogaster,* and its rearing method has been established. The egg‐to‐adult development time is approximately 13 days (Emiljanowicz et al., 2014).

### 2.2 Sampling and strains

We conducted field sampling of *D. suzukii* in 12 locations along urban–rural gradients across Tokyo and Chiba prefectures (Japan) during May–August 2019 and May–June 2020 (see Table S1 for details). Each location was more than 5 km from each other; thus, individuals collected from each location were considered different local populations. Pre‐mature and mature fruits of cherry blossoms, mulberries, and bayberries were collected at each location and placed under laboratory conditions (approximately 25°C) until the adults of *D. suzukii* emerged from the fruits. Adults that emerged were sexed, and a single female and male that emerged were enclosed in a vial (φ28 mm, 100 mm in height) filled with food medium to establish the isofemale line. Two to four isofemale lines were established for each local population. Prior to the experiment described below, the flies were reared for at least three generations under a constant environment (12L12D, 25°C) to remove the genetic variation within lines and standardize environmental and maternal effects. The food medium used was Jazz Mix *Drosophila* Food (Fisherbrand; Thermo Fisher Scientific) or Formula 4–24 Instant *Drosophila* Medium (Carolina Biological Supply Company). The former medium was used for experiments to determine the critical thermal maximum and minimum, and the latter was used for experiments on diurnal activity patterns. Inactive yeast (ca. 6 g) was added to the medium during the experiments to improve fly growth.

### 2.3 Population classification

The urbanization index (UI) for each sampling location was calculated based on satellite images. The UI reflects the dominance of vegetation. Satellite images taken on a sunny day (August 5, 2015) of Landsat 8 OLI band 5 (845–884 nm) and band 7 (2100–2300 nm) were obtained on a LandBrowser platform (National Institute of Advanced Industrial Science and Technology). The UI was calculated using the values of bands 5 and 7 in accordance with Kawamura et al. (1998): (band 7 − band 5)/(band 7 + band 5). The values of bands 5 and 7 for each location were calculated by averaging within a 5 km radius from the sampling site, excluding the sea area. Based on the estimated UI, 12 populations were classified into rural and urban populations. In the present study, four populations with UI < −0.35 and eight populations with UI > −0.35 were classified as rural and urban populations, respectively. Here UI < −0.35 represents that vegetation such as forest and agricultural land occupy half of the area and a region with UI > −0.35 is taken up more than half of the area by buildings and pavement roads.

### 2.4 Genetic difference in thermal tolerance

To measure the upper and lower thermal tolerance, we used temperature ramping assays to assess the critical thermal minimum (CT_min_) and maximum (CT_max_), which estimate the temperatures at which muscular coordination is lost (Jørgensen et al., 2019; MacLean et al., 2017). These assays are assumed to yield ecologically relevant performance limits and measure heat and cold tolerance (Bowler & Terblanche, 2008). Flies were placed into individual 0.5 ml microtubes without using CO_2_ anesthesia, which were closed with a cotton bud. Individuals with torn (regarded as old) or damaged wings were not used for the experiment. Temperatures were manipulated using an Applied Biosystems MiniAmp Thermal Cycler and were gradually increased (from 25 to 40°C) or decreased (from 25 to 3°C) at 1°C every 2 min (Enriquez & Colinet, 2019; Piyaphongkul et al., 2012). The temperatures at which a fly lost its ability to move after tapping on the vial were scored as CT_min_ and CT_max_, respectively. Eight urban and four rural populations were used in the experiment (see Table S1 for details). Two isofemale lines from each population were tested as replicates. The number of individuals tested per population for each thermal tolerance assay ranged from 20 to 57 for each sex. After the temperature ramping assays, we measured thorax width and wing length as proxies for body size. For the wing length, the distance between the end of the first longitudinal vein and the junction of the third longitudinal vein and the anterior crossvein was used.

### 2.5 Plastic changes in thermal tolerance

To measure the plastic changes in CT_min_ and CT_max_, flies were exposed to short‐term hardening treatments. We placed sexually mature flies individually in 0.5 ml microtubes without the use of CO_2_ anesthesia. Individuals with torn (regarded as old) or damaged wings were not used in the experiment. The tubes were sealed with wet cotton buds to avoid desiccation during hardening. Individuals in tubes were exposed to 3 or 32°C in an Applied Biosystems MiniAmp Thermal Cycler for 2 h and recovered at 25°C for 1 h before assessing CT_min_ or CT_max_ based on a previous study (Nyamukondiwa et al., 2011). As a control for each treatment, separate control treatments (25°C for 2 h followed by 1 h at 25°C) were performed simultaneously. After recovering, flies were exposed to ramping assays to demonstrate CT_min_ and CT_max_, respectively. The procedures were identical, except that the initial temperature was 12°C for the assays of CT_min_ and 35°C for CT_max_ to minimize the effect of acclimation to temperature exposure during assays on flies. For these treatment experiments, flies from two urban and two rural population were used (Table S1). Only one isofemale line per population was tested. We tested a range from 32 to 50 females and males per population for each thermal tolerance assay.

### 2.6 Daily activity pattern and the effect of light stress at night

We constructed a rearing experiment to examine daily activity patterns and the effects of artificial light at night. Isofemale lines from four urban and four rural populations were used (Table S1), and only one isofemale line per population was tested. To obtain imagos for the experiment, 30 females and 10 males were placed in a vial with a food medium and enclosed for 24 h in a constant environment to allow them to lay eggs. Immediately after enclosure, the females and males were removed, and eggs in the vials were exposed to two different environments with and without light at night in an incubator. For both environments, the air temperature was constant at 22°C and the light cycle was a 12:12 h light–dark cycle. For environments with and without light at night (ALAN), the light intensity during the dark period was approximately 0 and 10 Lx, respectively, whereas the light intensity during the day was approximately 2500 Lx (equivalent to an overcast day) (McLay et al., 2017).

Activity assays were performed using the Drosophila Activity Monitoring System (DAM5; Trikinetics Inc.). Each insect was detected by an infrared interruption method, and daily locomotion was recorded at 10‐min intervals using computer software (DAMSystem3 Software; Trikinetics Inc.). The females of the experimental generation were lightly anesthetized using CO_2_ and placed in an air‐vented transparent plastic straw (φ6 mm, 100 mm in length) plugged at one end with standard yeast‐agar‐sucrose and NaCl‐supplemented media and a lubber cap and the other end with a paper lid. The activity of flies was measured for 24 h in the environment in which they were reared.

### 2.7 Statistical analysis

All data analyses were conducted using R software v. 3.6.1. Differences in thermal tolerance and body size between the urban and rural populations were analyzed using generalized linear mixed models (GLMMs) with the *lmer* function from the “*lme4*” package (Bates et al., 2015). The models were constructed separately for CT_min_, CT_max_, thoracic breadth, and wing length as response variables. We included the fixed effects and two‐way interactions of urbanization type (urban or rural) and sex (female or male). We included both population IDs in each urbanization type and isofemale line IDs in each population as random factors (nested random factors). To avoid nested structure of random effects, we also conducted the GLMM with UI of each population IDs and sex as the fixed effects and line IDs in a population as a random effect.

To explore the effects of heat/cold exposure treatment on urban and rural populations, we applied GLMM with CT_min_/CT_max_ as the dependent variable. In each model, we included the treatment (heat or cold), urbanization type (urban or rural) and sex (female or male) as fixed effects and two‐way interactions of them. We included a population ID as random factors.

To extract the index of the pattern of daily activity, principal component analysis (PCA) was performed using data on log‐transformed locomotion counts per 1 h (24 variables). Then, the principal components with a contribution in total variance higher than 24/100 were used as the response variables. The effects of artificial light at night and urbanization type on each principal component were tested using a GLMM. In each model, we included the main and interaction effects of treatment (control or ALAN treatment) and urbanization type (urban or rural). The population ID in each urbanization type was included as a random effect.

Values are shown as the standard error of the mean, unless otherwise indicated. To test significance of each fixed effect, we ran Wald *χ* ^2^ tests by the *ANOVA* function in the “*car*” package in *R*.

## 3 RESULTS

### 3.1 Genetic difference in thermal tolerance

The thorax width did not significantly differ between urban (female: 0.96 ± 0.003 mm, male: 0.85 ± 0.003 mm) and rural populations (female: 0.95 ± 0.005 mm, male: 0.85 ± 0.004 mm, Tables S2). Likewise, no difference was found in the wing length between urban (female: 1.58 ± 0.004 mm, male: 1.41 ± 0.004 mm) and rural populations (female: 1.56 ± 0.006 mm, male: 1.40 ± 0.006 mm, Tables S3). The effect of urbanization type on thorax width differed between sexes (*χ* ^2^ = 4.0, *p* = .046; see Tables S3 for details).

**FIGURE 1:**
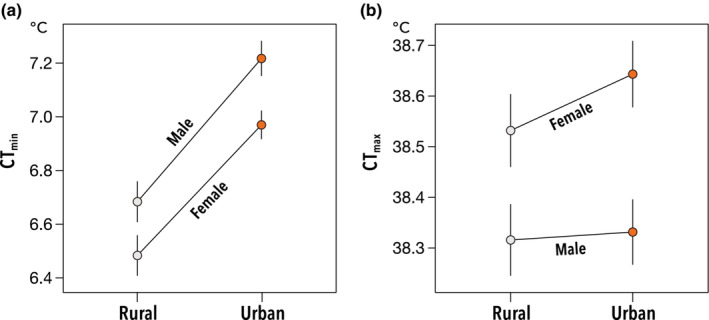
Genetic difference of CT_min_ (a) and CT_max_ (b) of urban and rural *Drosophila suzukii* populations

The mean CT_min_ of individuals derived from urban populations was clearly higher than those derived from rural population in both sexes (Figure 1a), though statistical significance was not found between urban and rural populations (urbanization type [U]: *χ* ^2^ = 2.68, *p* = .1; sex [S]: *χ* ^2^ = 14.8, *p* < .001; U × S: *χ* ^2^ = 0.2, *p* = .66; see also Table S4). Note that, the effect of UI on CT_min_ was significant in GLMM with isofemale line ID as only a single random effect (UI: *χ* ^2^ = 4.98, *p* = .026; sex: *χ* ^2^ = 11.03, *p* = .001; UI × sex: *χ* ^2^ = 0.021, *p* = .88). In contrast, we did not detect a difference in CT_max_ between urban and rural populations (*χ* ^2^ = 0.03, *p* = .85; Table S5 and Figure 1b). The effect of UI on CT_max_ was not also significant (UI: *χ* ^2^ = 0.15, *p* = .70; sex: *χ* ^2^ = 3.27, *p* = .071; IU × sex: *χ* ^2^ = 1.29, *p* = .25).

### 3.2 Plastic changes in thermal tolerance

**FIGURE 2:**
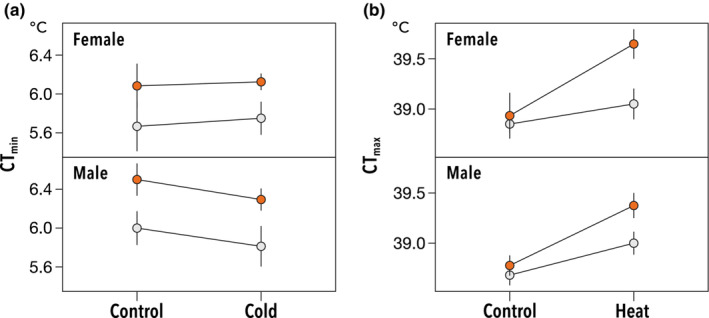
Effect of short‐term hardening treatment on thermal tolerances of urban and rural *Drosophila suzukii* populations. CT_min_ was measured after the exposure to 25°C (control) or 3°C (cold) (a). CT_max_ was measured after the exposure to 25°C (control) or 32°C (heat) (b).

For CT_min_, no significant difference was found between treatments (*χ* ^2^ = 0.26, *p* = .61; Table S6 and Figure 2a). Consistent with the experiment on genetic differences in cold tolerance, urban populations exhibited lower cold tolerance (*χ* ^2^ = 12.5, *p* < .001). We did not detect a significant effect of sex on the CT_min_ (Table S6). The CT_min_ was not influenced by any interaction effect between the variables. For CT_max_, we found no significant difference in heat tolerance among urban and rural populations (Table S7 and Figure 2b) and a significant effect of sex, both consistent with the experiment of genetic differences in heat tolerance. However, CT_max_ significantly increased with heat hardening, irrespective of the urbanization level (*χ* ^2^ = 27.0, *p* > .001). In addition, there was a significant interaction effect between urbanization type and treatment (*χ* ^2^ = 5.8, *p* = .015), indicating a strong response in heat tolerance against heat hardening in individuals of urban populations than those of the rural populations of *D. suzukii*, which have a greater ability to improve heat tolerance in response to high‐temperature exposure.

### 3.3 Daily activity and the effect of light stress at night

**FIGURE 3:**
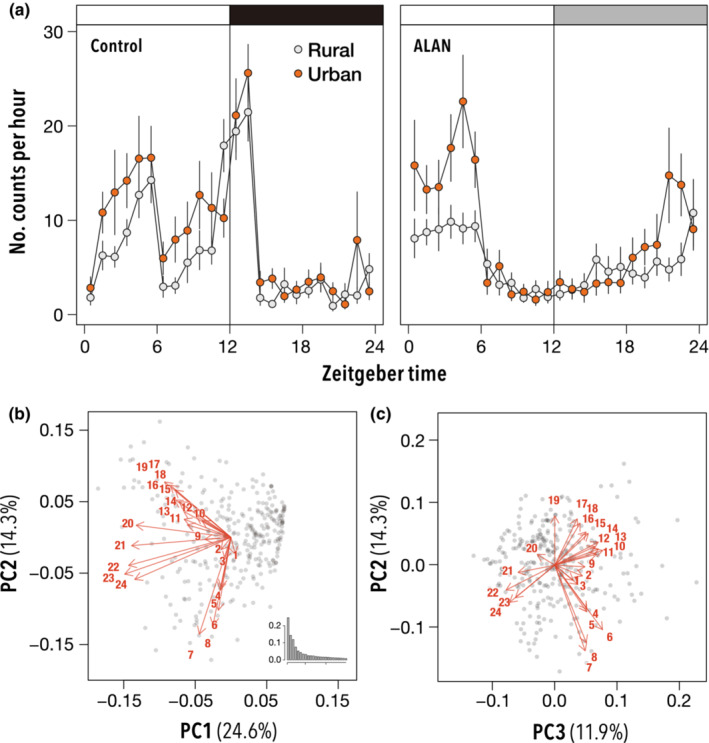
Effect of ALAN on diurnal activity patterns of females of urban and rural *Drosophila suzukii* populations. Number of counts per hour of urban and rural populations under control and ALAN treatments (a), first and second principal components and histogram of eigenvalues (b), and second and third principal components (c). The inset indicates the eigenvalues of the analysis.

Both urban and rural populations exhibited bimodal patterns of activities with peaks in the morning (1:00–6:00) and evening (12:00–14:00) in the condition without ALAN (Figure 3A). In contrast, under ALAN conditions, they exhibited unimodal patterns, where the morning peaks were relatively higher than those under the treatment (non‐ALAN) condition, and the evening peaks were strongly diminished. In addition, night‐time activity levels remained high under ALAN conditions compared with those under non‐ALAN conditions.

**FIGURE 4:**
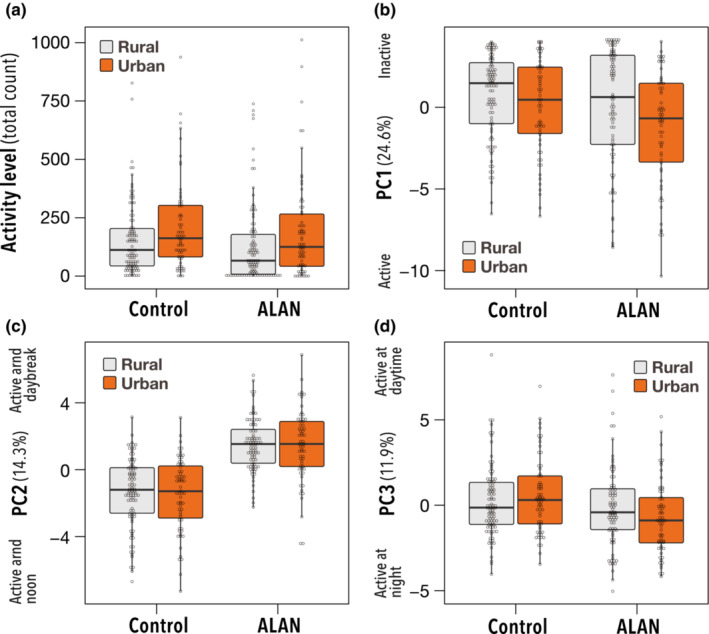
Effects of ALAN during development on diurnal activity patterns of adult female *Drosophila suzukii* individuals of urban and rural populations. Activity level (a), PC1 (b) PC2 (c), and PC3 (d).

The total activity of urban populations was slightly greater than that of rural populations (*χ* ^2^ = 3.72, *p* = .053; Figure 4a) and declined significantly with ALAN (*χ* ^2^ = 262, *p* < .001). For the urban population, the decline in total activity under ALAN was less than that for the rural population (*χ* ^2^ = 23.1, *p* < .001). We performed PCA using activities every hour for a day. The analysis revealed that much of the variation in activity could be explained by the first, second, and third principal components (PC1, PC2, and PC3) which explained more than 10% of the total variance (cumulative approximately 50%; Figure 3). PC1 accounted for more than 24.6% of the total variance, which was interpreted as the level of activity at night. PC1 was consistently influenced by activity level, especially during the night, with smaller values indicating higher activity levels. PC2 counts for 14.3% of the variance. PC2 indicated the contrast of activity level around noon and around midnight, that is, the rhythm and pattern of daily activity. Individuals with high PC2 scores can be described as active at noon (6:00–8:00) relative to midnight (17:00–19:00), and vice versa. PC3 accounts for 11.9% of the total variance. PC3 also represented the rhythm and pattern of daily activities. Individuals with high PC3 scores indicated a strong activity level around sunrise (22:00–24:00) relative to around sunset (10:00–14:00).

We found a slight but significant difference in the PC1 score between the two light treatments (*χ* ^2^ = 6.07, *p* = .014; Figure 4b), indicating that ALAN increased activity at night in individuals exposed to the ALAN treatment. No difference in PC1 was found between urban and rural populations (*χ* ^2^ = 1.27, *p* = .26). The effect of ALAN treatment on PC1 tended to be larger in the urban than rural population, although we did not find a significant interaction effect between ALAN treatment and urbanization type or on PC1 score (*χ* ^2^ = 0.73, *p* = .39). For PC2, individuals exposed to ALAN exhibited higher scores than those in the control treatment, indicating that ALAN enhanced activity at midnight (*χ* ^2^ = 161.3, *p* < .001; Figure 4c). There were no significant effects of urbanization type and the interaction term between urbanization type and treatment on the PC2 score (urbanization type: *χ* ^2^ = 0.15, *p* = .70; interaction: *χ* ^2^ = 0.009, *p* = .92). PC3 was higher in individuals exposed to ALAN than in those in the control group (*χ* ^2^ = 10, *p* = .014; Figure 4d), indicating that ALAN enhanced the degree of activity around sunrise. This pattern was greater in urban than rural populations (interaction effect of urbanization type and treatment: *χ* ^2^ = 4.4, *p* = .035).

## 4 DISCUSSION

Urbanization alters several aspects of the biotic and abiotic environment. Although some studies have described rapid phenotypic changes in response to urban environmental changes (Beasley et al., 2018; Grunst et al., 2020; Su et al., 2021), studies that distinguish between genetic and plastic changes are limited because of difficulties in controlling genetic and environmental effects and in removing maternal effects. In the present study, we focused on thermal and luminary environments to investigate both genetic and plastic changes in the thermal tolerance and daily activity patterns of *D. suzukii*. We detected an evolution in thermal tolerance and diurnal activity patterns in urban populations. In addition, our results revealed that urban populations evolved greater abilities to respond to heat exposure and ALAN. These results suggested that rapid phenotypic changes in urban organisms are shaped by a combination of genetic and environmental factors and their interaction effects.

Heat in cities is critical for organisms (Hall & Warner, 2018), and can induce thermal adaptation. Since cities are relatively warmer than rural areas (Oke, 1982; Stewart & Oke, 2012; Tran et al., 2017), cold tolerance is expected to be weaken in city population because it may be costly to maintain cold tolerance (Diamond et al., 2018). In the present study, urban populations of *D. suzukii* showed reduced cold tolerance compared to rural populations, suggesting that lower cold tolerance evolved rapidly along rural–urban gradient. Ectotherms often achieve thermal tolerance by changing their body size (Calosi et al., 2008). However, body size (thorax breadth and wing length) did not differ between the urban and rural populations for both sexes. Taken together, the evolution of lower cold tolerance may be achieved via changes in physiological and molecular properties rather than body size.

Cities provide conditions with higher temperatures, which possibly promote evolution with higher heat tolerance. Indeed, some studies have reported improved heat tolerance in urban organisms (Martin et al., 2019). In contrast, our results showed that urban populations tended to have a higher heat tolerance, but this was not remarkable. Several mechanisms may explain this evolutionary stagnation: (1) Heat tolerance is on the way to evolution in this species. Since population size in urban environments is generally small, the standing genetic variation might be scarce or adaptive mutation did not occur to evolve sufficiently (Diamond & Martin, 2020); (2) the evolutionary development of plasticity to improve heat tolerance may hinder or evolve prior to the evolution of heat tolerance itself (Diamond & Martin, 2016). Since individuals in urban populations showed greater plasticity to improve heat tolerance than those in rural populations, our results suggested that the evolution of greater plasticity of thermal tolerance may be related to the evolutionary stagnation of heat tolerance itself. If an individual with genetically high heat tolerance emerges in the future, such an allele can be fixed in urban populations, leading to the evolution of heat tolerance itself (Price et al., 2003). Our system may provide evidence for the plasticity‐first hypothesis in natural populations.

Our locomotion activity assay showed a greater amount of total activity in urban populations than in rural populations, indicating that urban populations evolved more actively, although analysis using principal components did not detect a significant difference in activity level. In contrast, the rhythm and pattern of daily activity did not differ between urban and rural populations, suggesting that the rhythm and pattern of daily activity did not evolve along the urbanization gradient. However, ALAN critically diminished the peak of activity around the onset of night, when fruit flies mainly forage, mate, and oviposit (Lin et al., 2014; Revadi et al., 2015). Our results suggested that ALAN leads to a decrease in food intake and disrupts opportunities for mating and oviposition. Consequently, ALAN may have a crucial influence on the survival and reproduction of this species. In addition, the levels of the ALAN effect were different between the urban and rural populations. In the ALAN condition, the activity of individuals of the urban population was enhanced immediately before light‐on relative to rural ones, indicating that individuals of urban populations managed to compensate for the decline in activity level due to ALAN. Our results also suggested that an evolutionary shift in activity patterns under ALAN conditions results in the expansion or shift of the temporal niche. The temporal niche at night may be relatively vacant, thus leading to relaxed feeding competition, avoidance of enemies, avoidance of heat stress during the daytime, etc. (Campera et al., 2019; Cunningham et al., 2019).

In summary, we investigated the genetic and plastic responses to urban environments in *D. suzukii* and revealed that the urban populations evolved in thermal tolerance and diurnal activity patterns, as well as artificial night light and heat, changed thermal tolerance and activity patterns. The present study highlights the relative contribution of multiple effects on organismal responses to rapid human‐induced environmental changes. To reveal the reproductive/ecological consequences in cities, future studies should examine the evolutionary and plastic responses of reproductive and ecological traits. Moreover, to demonstrate the ubiquity of urban evolution, we should conduct similar experiments for several sets of urban–rural gradients.

## AUTHOR CONTRIBUTIONS

**Ayame Sato:** Conceptualization (equal); data curation (equal); formal analysis (equal); investigation (equal); validation (equal); visualization (lead); writing – original draft (lead). **Yuma Takahashi:** Conceptualization (equal); data curation (supporting); formal analysis (supporting); funding acquisition (lead); investigation (equal); project administration (lead); supervision (lead); validation (supporting); visualization (supporting); writing – original draft (supporting); writing – review and editing (supporting).

## CONFLICT OF INTEREST

The authors declare no competing interests.

## Supporting information


Table S1.

## Data Availability

Data are available online (Sato & Takahashi, 2022, Dryad Digital Repository: doi:10.5061/dryad.gqnk98sq5).
