# Supplementary material for: Responses in thermal tolerance and daily activity rhythm to urban stress in Drosophila suzukii
Source: Ecol Evol. 2022 Dec 12;12(12):e9616. doi: 10.1002/ece3.9616 (PMC9744627; doi:10.1002/ece3.9616)
Supplement: Supplementary file 1 — Table S1. [file ECE3-12-e9616-s001.docx]

# Tables

**Table S1.** Populations of *Drosophila suzukii* used for the experiments.

| population | latitude | longitude | UI | UD | urbanization type | experiment |
| --- | --- | --- | --- | --- | --- | --- |
| ASU | 139.739 | 35.7505 | −0.1382594 | 0.332 | urban | DA |
| ICH | 140.06024 | 35.511902 | −0.2024096 | 0.121 | urban | GT, PT |
| KAS | 140.198939 | 35.399729 | −0.4017793 | 0.018 | rural | GT |
| KIM | 139.883103 | 35.343964 | −0.2448924 | 0.104 | urban | GT |
| KOK | 139.743351 | 35.681779 | −0.1587633 | 0.336 | urban | GT, PT |
| KUR | 140.089819 | 35.287714 | −0.4091599 | 0.009 | rural | GT, PT, DA |
| NOG | 140.1129 | 35.4631 | −0.3463065 | 0.046 | rural | DA |
| RES | 140.244 | 35.4785 | −0.3734124 | 0.026 | rural | GT, DA |
| RYO | 139.989553 | 35.44913 | −0.2741263 | 0.076 | urban | GT |
| SAK | 140.224841 | 35.714279 | −0.3219174 | 0.078 | urban | GT |
| SEA | 139.749508 | 35.607795 | −0.1391502 | 0.294 | urban | DA |
| SHO | 140.279418 | 35.516255 | −0.3395542 | 0.05 | urban | GT |
| SOG | 140.12812 | 35.571898 | −0.1727152 | 0.17 | urban | GT |
| SUM | 139.804 | 35.7126 | −0.1252607 | 0.338 | urban | DA |
| TEN | 140.547796 | 35.705689 | −0.3658095 | 0.05 | rural | GT, PT, DA |
| YAY | 140.103849 | 35.628102 | −0.1554236 | 0.196 | urban | GT, DA |

GT: test for genetic differences in thermal tolerance; PT: test for plastic differences in thermal tolerance; DA: test for diurnal activity.

**Table S2.** Results of GLMM analysis testing for the genetic difference of thorax width between urban and rural populations of *Drosophila* *suzukii*.

| Source | χ^2^ | *df* | *P* |
| --- | --- | --- | --- |
| Urbanization type (urban/rural) | 0.002 | 1 | 0.96 |
| Sex (female/male) | 1041.9 | 1 | < 0.001 |
| Urbanization type × Sex | 4.0 | 1 | 0.046 |

**Table S3.** Results of GLMM analysis testing for the genetic difference of wing length between urban and rural populations of *Drosophila* *suzukii*.

| Source | χ^2^ | *df* | *P* |
| --- | --- | --- | --- |
| Urbanization type (urban/rural) | 0.61 | 1 | 0.44 |
| Sex (female/male) | 1593.9 | 1 | < 0.001 |
| Urbanization type × Sex | 2.89 | 1 | 0.09 |

**Table S4.** Results of GLMM analysis testing for the genetic difference of CT_min_ between urban and rural populations of *Drosophila suzukii*.

| Source | χ^2^ | *df* | *P* | |
| --- | --- | --- | --- | --- |
| Urbanization type (urban/rural) | 2.68 | 1 | 0.10 |  |
| Sex (female/male) | 14.8 | 1 | < 0.001 |  |
| Urbanization type × Sex | 0.52 | 1 | 0.47 |  |

**Table S5.** Results of GLMM analysis testing for the genetic difference of CT_max_ between urban and rural populations of *Drosophila suzukii*.

| Source | χ^2^ | *df* | *P* |
| --- | --- | --- | --- |
| Urbanization type (urban/rural) | 0.038 | 1 | 0.85 |
| Sex (female/male) | 18.1 | 1 | < 0.001 |
| Urbanization type × Sex | 0.52 | 1 | 0.47 |

**Table S6.** Results of GLMM analysis testing for the effect of cold/heat exposure on CT_min_ in urban and rural populations of *Drosophila suzukii*.

| Source | χ^2^ | *df* | *P* |
| --- | --- | --- | --- |
| Treatment (control/cold-hardened) | 0.26 | 1 | 0.61 |
| Urbanization type (urban/rural) | 12.5 | 1 | < 0.001 |
| Sex (female/male) | 3.2 | 1 | 0.074 |
| Treatment × Urbanization type | 0.014 | 1 | 0.90 |
| Treatment × Sex | 1.0 | 1 | 0.31 |
| Urbanization type × Sex | 0.15 | 1 | 0.70 |

**Table S7.** Results of GLMM analysis testing for the effect of cold/heat exposure on CT_max_ in urban and rural populations of *Drosophila suzukii*.

| Source | χ^2^ | *df* | *P* |
| --- | --- | --- | --- |
| Treatment (control/heat-hardened) | 27.0 | 1 | < 0.001 |
| Urbanization type (urban/rural) | 0.41 | 1 | 0.52 |
| Sex (female/male) | 4.0 | 1 | 0.045 |
| Treatment × Urbanization type | 5.8 | 1 | 0.016 |
| Treatment × Sex | 0.070 | 1 | 0.79 |
| Urbanization type × Sex | 0.51 | 1 | 0.48 |
